# Specific Diurnal EMG Activity Pattern Observed in Occlusal Collapse Patients: Relationship between Diurnal Bruxism and Tooth Loss Progression

**DOI:** 10.1371/journal.pone.0101882

**Published:** 2014-07-10

**Authors:** Shigehisa Kawakami, Yohei Kumazaki, Yosuke Manda, Kazuhiro Oki, Shogo Minagi

**Affiliations:** Department of Occlusal and Oral Functional Rehabilitation, Graduate School of Medicine, Dentistry and Pharmaceutical Sciences, Okayama University, Okayama, Japan; University of Southern California, United States of America

## Abstract

**Aim:**

The role of parafunctional masticatory muscle activity in tooth loss has not been fully clarified. This study aimed to reveal the characteristic activity of masseter muscles in bite collapse patients while awake and asleep.

**Materials and Methods:**

Six progressive bite collapse patients (PBC group), six age- and gender-matched control subjects (MC group), and six young control subjects (YC group) were enrolled. Electromyograms (EMG) of the masseter muscles were continuously recorded with an ambulatory EMG recorder while patients were awake and asleep. Diurnal and nocturnal parafunctional EMG activity was classified as phasic, tonic, or mixed using an EMG threshold of 20% maximal voluntary clenching.

**Results:**

Highly extended diurnal phasic activity was observed only in the PBC group. The three groups had significantly different mean diurnal phasic episodes per hour, with 13.29±7.18 per hour in the PBC group, 0.95±0.97 per hour in the MC group, and 0.87±0.98 per hour in the YC group (*p*<0.01). ROC curve analysis suggested that the number of diurnal phasic episodes might be used to predict bite collapsing tooth loss.

**Conclusion:**

Extensive bite loss might be related to diurnal masticatory muscle parafunction but not to parafunction during sleep.

**Clinical Relevance: Scientific rationale for study:**

Although mandibular parafunction has been implicated in stomatognathic system breakdown, a causal relationship has not been established because scientific modalities to evaluate parafunctional activity have been lacking.

**Principal findings:**

This study used a newly developed EMG recording system that evaluates masseter muscle activity throughout the day. Our results challenge the stereotypical idea of nocturnal bruxism as a strong destructive force. We found that diurnal phasic masticatory muscle activity was most characteristic in patients with progressive bite collapse.

**Practical implications:**

The incidence of diurnal phasic contractions could be used for the prognostic evaluation of stomatognathic system stability.

## Introduction

Bruxism has caused great concern as a cause of periodontal tissue destruction. However, the relationship between bruxism and tooth loss is not clear, despite the great efforts of epidemiological surveys such as the Study of Health in Pomerania [1]. Increased nocturnal masticatory muscle activity has been thought to cause occlusal overloads, thereby causing clinical complications for teeth and prostheses [2]. Problems possibly related to masticatory muscle forces are periodontal disease and tooth fracture. However, Houston et al. [3] suggested that there is weak or absent correlation between periodontal disease and bruxism, and between bruxism and occlusal status. Bernhardt et al. [1] reported that bruxism, as disclosed on questionnaires, as well as occlusal wear into dental hard tissues, protrusive contacts, and elongation of teeth, were not associated with probing depth.

One possible explanation for these conflicting reports might be overemphasis on nocturnal bruxism. The reliability of questionnaires as a method of recording bruxism incidence is another possible factor in these inconsistencies. In their review, Manfredini et al. [4] found that most internal flaws arose because data were derived from studies based on self-reported questionnaires, most of which contained a single bruxism question within a comprehensive history questionnaire, raising concerns about within-study specificity and between-study homogeneity of criteria to diagnose bruxism.

To reveal the effect of masticatory force on stomatognathic system issues, exact evaluation of the quality and quantity of exerted forces is necessary, not only during sleep but also during waking hours. Although a few studies have reported diurnal electromyogram (EMG) activity [5], [6], most EMG analyses have focused on sleep bruxism. In adolescence, diurnal clenching has been regarded as a possible risk factor for intermittent locking [7]. The purpose of this study was to qualitatively and quantitatively evaluate the masseter muscle EMG activity of occlusal collapse patients while awake and asleep.

## Materials and Methods

### Subjects

Three subject groups were used in this study: the progressive bite collapse group (PBC group), the matched control group (MC group), and the young control group (YC group).

PBC group patients were selected according to the criteria described below from among 415 outpatients of four dentists in the department of occlusion and denture prosthodontics at Okayama University Hospital. Six patients who met the criteria were consecutively enrolled during the period from October 2012 to January 2013. The subjects’ occlusal contact was evaluated according to the Eichner Index (EI) [8], [9], based on existing contact points between natural teeth in the maxilla and mandible in the bilateral premolar and molar regions. According to the EI, dentition restored with a fixed partial denture was considered natural dentition. Subgroup EI definitions are as follows [8]. Group A1 has no missing teeth in the mandible or maxilla, group A2 has at least one missing tooth in either the mandible or the maxilla, and group A3 has at least one missing tooth in both the mandible and the maxilla. Groups B1, B2, and B3 have posterior occlusal contact(s) in three, two, and one zone(s), respectively; group B4 has occlusal contact(s) in the anterior region only. Group Cl has at least one tooth in both the mandible and the maxilla without any occlusal contact, group C2 has at least one tooth without contact in either the mandible or the maxilla, and group C3 is fully edentulous in both arches. The PBC group included four men and two women (mean age, 71.4±8.0 years). Inclusion criteria for the PBC group were: (1) B4 EI classification, (2) posterior teeth remaining in the mandible and/or maxilla, (3) loss of one or more teeth from the remaining occlusal antagonistic tooth pairs in the past year, (4) history of needing repeated denture adjustment for more than 1 year because of pain in supporting tissues, and (5) agreement to participate in this study. Patients who fulfilled all five criteria were selected as the PBC group. All patients in the PBC group wore removable partial dentures.

The MC group included individuals age- and gender matched with the PBC group. The MC group consisted of four men and two women (mean age, 71.5±10.4 years) in EI category A or B1–B3. These patients visited the clinic to receive regular check-ups. Inclusion criteria for the MC group were: (1) A or B1–B4 EI classification, (2) no tooth loss in the past year, (3) no need for denture adjustment for more than 1 year, and (4) agreement to participate in this study. Although two subjects in the MC group had removable denture prosthesis, neither had needed denture adjustment in the past year. Patients fulfilling the above four criteria were selected from among the outpatients of one dentist from February to April 2013 to match the PBC group in age and gender. Individuals who fulfilled the above criteria were consecutively selected from among the outpatients to avoid selection bias.

In the MC group, the mean number of remaining teeth was 23.2±4.9 (range: 16–28) and the mean number of remaining occlusal antagonistic tooth pairs was 10.5±3.2 (range: 6–14). As a general qualitative finding, frequent and continuous phasic diurnal EMG activity was most evident in the PBC group, as shown in Figure 2.

Figure 3 shows the frequency of diurnal phasic episodes, using a threshold of 20% EMG, summarized according to the duration of each episode. Diurnal phasic episodes of long duration, lasting up to 1 min, were most frequently observed in the PBC group. By contrast, only a few long phasic episodes were observed in the MC and YC groups. Moreover, phasic episodes lasting longer than 2 min were observed in the PBC group, whereas no episodes longer than 40 s were observed in the MC or YC groups.

The YC group consisted of gender-matched but young subjects. Four men and two women (mean age, 22.3±0.8 years) with full dentition except for wisdom teeth were enrolled. They were classified as A1 according to the EI. The YC group was recruited from Okayama University Dental School students. Of 11 male and 22 female students who agreed to participate, three men and five women were excluded because of a history of orthodontic treatment or group I classification according to the Research Diagnostic Criteria for Temporomandibular Disorders (RDC/TMD). Four men and two women with group 0 RDC/TMD classification were randomly selected, using randomized numbering, from the remaining eight men and 15 women.

The sample size of six subjects in each group was determined from the results of our preliminary measurements on three equivalently categorized subjects in the PBC and YC groups. The calculation was made using α = 0.05, (1-β) = 0.8 and effect size = 1.7, using G*Power 3.1.7 [10].

### EMG data recording

The ambulatory EMG recording hardware consisted of an analog signal processing and differential amplification integrated hybrid circuit (NB-6201HS; Nabtesco Co., Kobe, Japan), which included a high-pass filter (10 Hz) and a low-pass filter (1000 Hz), and a two-channel digital recorder (ICR-PS004M; Sanyo Electric Co., Ltd., Osaka, Japan). An EMG of the left masseter muscle was recorded using differential surface electrodes composed of three disposable Ag/AgCl surface electrodes (6×15 mm, Vitrode F-150S; Nihon Kohden Corp., Tokyo, Japan) with center-to-center distances of 15 mm. The electrodes and cables were secured to the buccal skin with thin biocompatible adhesive tape (Cathereep FS 1010; Nichiban Co. Ltd., Tokyo, Japan).

Subjects were instructed to perform maximal voluntary clenching (MVC) three times for 2 s at intervals of 2 s. For EMG standardization, signal levels of 20% MVC and 5N were used as thresholds in signal analysis. Subjects were instructed to gently and gradually bite an occlusal force detector on the left first molar five times until the bite force reached 5N.

To distinguish EMG activity during speech, a voice-operated trigger switch (VOX) was used with a condenser microphone attached to the neck skin adjacent to the larynx [11]. The VOX generated a signal voltage for each phonation, which was recorded as a negative electrical trigger signal at the beginning and a positive electrical trigger signal at the end of the phonation.

To eliminate the first night effect, subjects were instructed to wear dummy electrodes and cables during sleep on the day before the real measurement.

Subjects were instructed to remove the VOX microphone from their neck skin just before sleeping.

### Data analysis

Recorded EMG data were processed off-line, filtered with a low-pass filter (500 Hz) and a notch filter (60 Hz), and then down sampled to 100 Hz. EMG signals accompanied by positive VOX signals were regarded as speech activities and excluded from subsequent analysis.

An EMG level of 20% MVC was adopted as the threshold for analysis of phasic, tonic, and mixed EMG activities, according to the standard reported by Velly-Miguel et al. [12]. Bruxism activities were analyzed according to the diagnostic cut-off criteria with 20% MVC threshold according to Lavigne et al. [13]: (1) more than four bruxism episodes per hour and (2) more than six bruxism bursts per episode and/or 25 bruxism bursts per hour of sleep. Because no sound recordings were made in the present study, Lavigne’s grinding sound criterion was not used. EMG activities during mastication were excluded from subsequent analysis.

The onset of sleep was defined as 10 min after the last VOX signal, because removing the microphone from the neck skin inevitably generated the last VOX signal. The strength of the EMG signal for 5 N biting was converted to % MVC for inter-individual comparison.

### Statistical analysis

Bruxism episodes per hour obtained from EMG data using the 20% MVC threshold were tested by two-way analysis of variance. The within-subject factor was the type of EMG episode: phasic, tonic, or mixed. The between-subject factor was experimental group: PBC, MC, or YC. When Mauchly's sphericity was violated in this analysis, the degree of freedom was adjusted by the Greenhouse-Gesser correction so that the F-ratio could be validly modulated. The interaction between factors was confirmed to be insignificant before the main effect of each factor was analyzed by the Bonferroni method as a post-hoc test. A significance level of 0.05 was adopted for all statistical tests, and two-sided tests were applied. Data analyses were performed using Predictive Analytic Software Statistics 17.0 (SPSS, Inc., Chicago, IL, USA).

Differences among PBC, MC, and YC groups in mean duration and mean burst strength of phasic episodes were evaluated using the Student’s t-test. The sample size necessary to attain statistical power was calculated in advance using G*Power 3.1.7. [10]. Post hoc power analysis was also performed to evaluate sample size adequacy. All subjects received an explanation of the nature and purpose of the study, and all provided their written, informed consent to participate in the study. The study protocol was approved by the ethics committee of Okayama University (No. 1646).

## Results

Subject characteristics are shown in Table 1. In the PBC group, the mean number of remaining teeth was 13.0±4.5 (range: 6–17) and the mean number of remaining occlusal antagonistic tooth pairs was 2.5±1.5 (range: 1–5).

**Table 1:** Clinical parameters and demographic characteristics of study subjects.

Subject	Sex	Age(years)	EichnerClassification	Dental Formula ofRemaining Teeth	Number of Remaining Teeth	Number of remaining occlusal antagonistic tooth pairs
PBC1	m	77	B4	654|3 4––––––––––––1|123	9	1
PBC2	m	70	B4	321|1 3––––––––––––76 321|12345 7	16	5
PBC3	m	78	B4	765 3|7––––––––––––3|	6	1
PBC4	m	63	B4	76 1|1 3456––––––––––––54321|123	16	3
PBC5	f	60	B4	7654321|––––––––––––21|12345	14	2
PBC6	f	78	B4	3|23 7––––––––––––7654321|123456	17	3
MC1	m	61	A1	7654321|1234567––––––––––––7654321|1234567	28	14
MC2	m	71	A3	765 21|1234567––––––––––––7654321|123456	25	11
MC3	m	61	A1	7654321|1234567––––––––––––7654321|1234567	28	14
MC4	m	84	B1	4 3|234 6––––––––––––65432|23456	16	6
MC5	f	84	B2	54321|12345––––––––––––7654321|123456	23	10
MC6	f	68	B3	54321|123456––––––––––––321|12345	19	8
YC1	m	23	A1	7654321|1234567––––––––––––7654321|1234567	28	14
YC2	m	22	A1	7654321|1234567––––––––––––7654321|1234567	28	14
YC3	m	23	A1	7654321|1234567––––––––––––7654321|1234567	28	14
YC4	m	23	A1	7654321|1234567––––––––––––7654321|1234567	28	14
YC5	f	21	A1	7654321|1234567––––––––––––7654321|1234567	28	14
YC6	f	22	A1	7654321|1234567––––––––––––7654321|1234567	28	14

**Figure 1:**
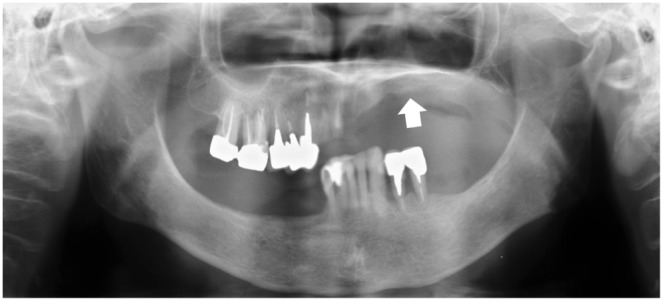
Typical panorama x-ray view of Eichner Index B4 patient. Note the absence of posterior occlusal support with highly resorbed alveolar bone (white arrow).

A typical panorama X-ray view from the PBC group is shown in Figure 1. Despite the remaining teeth, molar occlusal support is completely lost and marked alveolar bone resorption in the denture-supporting area is observed.

**Figure 2:**
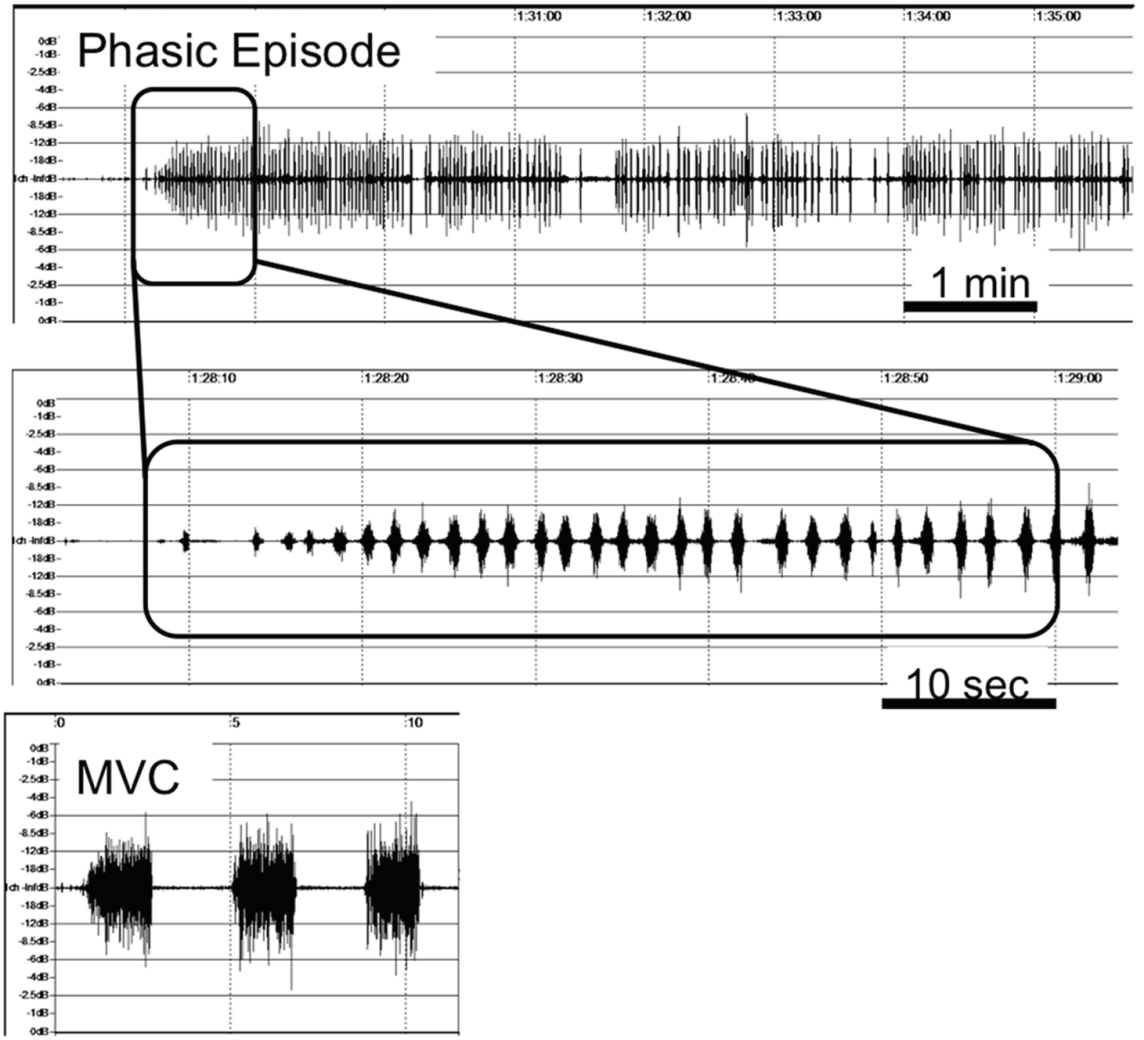
Typical diurnal phasic EMG activity observed in the PBC group. MVC, EMG activity during maximal voluntary clenching.

**Figure 3:**
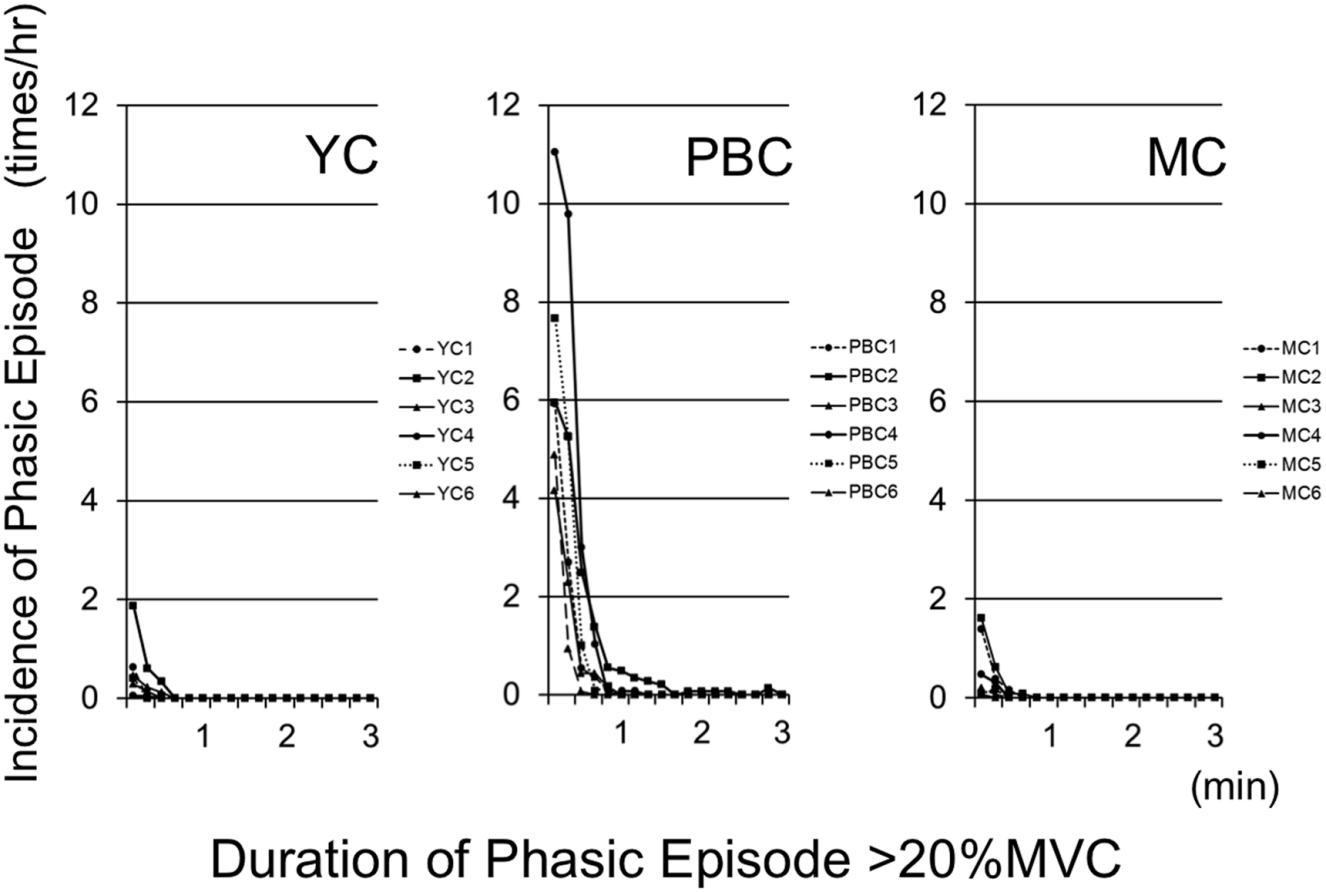
Incidence of phasic episodes relative to the duration of each episode (threshold: >20% MVC). Diurnal phasic episodes of long duration were most frequently observed in the PBC group. Note that all the subject, in MC and YC groups, showed episodes less than twice per hour.

**Figure 4:**
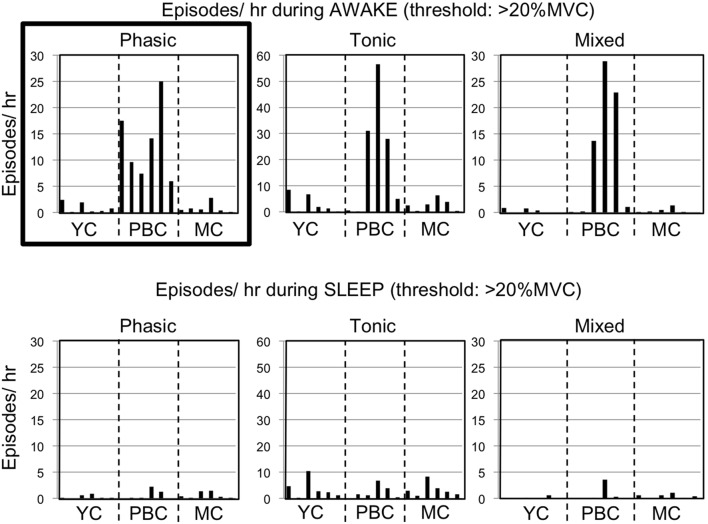
Incidence of phasic, tonic, and mixed episodes >20% MVC for YC, PBC, and MC groups. Note that, during waking hours, all the subjects in PBC group showed high prevalence of phasic episodes. On the other hand, despite the fact that most of the subjects in PBC group showed high prevalence of tonic and mixed episodes, some of the subjects in PBC group showed only few episodes of these types, which seemed to be almost equivalent with those in YC and MC groups. Incidence of phasic episodes during sleep for PBC, MC and YC groups were remarkably low compared to those in waking hours (*p* = 0.008).

Figure 4 shows the results of diurnal and nocturnal bruxism analysis from the EMG recordings, based on the definition of bruxism by Velly-Miguel et al. [12] using a threshold of 20% MVC.

In the PBC group, phasic, tonic, and mixed episodes tended to occur more frequently than in the MC and YC groups. However, three subjects in the PBC group showed only a few tonic and mixed episodes. During waking hours, the mean number of phasic episodes per hour with a threshold of 20% MVC were 13.29±7.18 in the PBC group, 0.95±0.97 in the MC group, and 0.87±0.98 in the YC group. In the PBC group, diurnal phasic episode frequency was in the range of 5.96–25.03 episodes/h.

By contrast, in the MC and YC groups, only a few diurnal phasic episodes were observed, with 0.07–2.38 episodes/h for the MC group and 0.10–2.82 episodes/h for the YC group. Episodes higher than 20% MVC were more frequently observed during waking hours than during sleep in the PBC group. The mean cumulative duration of phasic episodes for the PBC group during waking hours was 15.46 min/h, whereas that during sleep was 0.50 min/h. The mean cumulative duration of diurnal phasic episodes higher than 20% MVC was only 0.66 min/h for the YC group and 1.04 min/h for the MC group.

Two-way analysis of variance was performed to evaluate the main effects of “group” (PBC, MC, or YC) and “episode” (phasic, tonic, or mixed). There was no intersection between “group” and “episode” (AWAKE, >20% MVC: F(2.09, 15.65) = 0.54, η^2^ = 0.02, *p* = 0.60; ASLEEP, >20% MVC: F(2.12, 15.92) = 0.65, η^2^ = 0.03, *p* = 0.54). Therefore, the effects of “group” and “episode” were subsequently evaluated.

The results of Bonferroni multiple comparisons and mean episodes/h for the six subjects in each group are shown in Table 2. For the main effect, “group”, only AWAKE (>20% MVC) showed a significant effect (F(2, 15) = 6.82, η^2^ = 0.35, *p* = 0.01). For the condition of ASLEEP (>20% MVC), no significant effect of “group” could be observed (ASLEEP, >20% MVC: F(2, 15) = 0.07, η^2^<0.01, *p* = 0.93). Tonic episodes (>20% MVC) were significantly more frequent than phasic or mixed during sleep. No significant differences were observed among the groups. By contrast, for muscle activity during waking hours (>20% MVC), the direction of the main effect was reversed. There were no significant differences in the incidence of different types of episode. Regarding the effect of “group”, the incidence of episodes in the PBC group was significantly higher than in the MC or YC groups.

**Table 2:** Mean number of episodes per hour.

	AWAKE (>20%MVC)			SLEEP (>20%MVC)		
	Phasic	Tonic	Mixed	Phasic	Tonic	Mixed
MC	0.95	3.06	0.35	0.3	3.56#	0.14
	(0.97)	(3.54)	(0.42)	(0.32)	(3.67)	(0.21)
PBC	13.29§	20.19§	11.13§	0.61	2.28#	0.66
	(7.18)	(22.45)	(12.65)	(0.90)	(2.54)	(1.42)
YC	0.87	2.72	0.35	0.63	3.35#	0.45
	(0.98)	(2.27)	(0.51)	(0.62)	(2.65)	(0.38)

Mean number (S.D.).

# *p*<0.05, vs Phasic and Mixed,

§ *p*<0.05, vs MC and YC.

(Bonferroni multiple comparison test).

**Figure 5:**
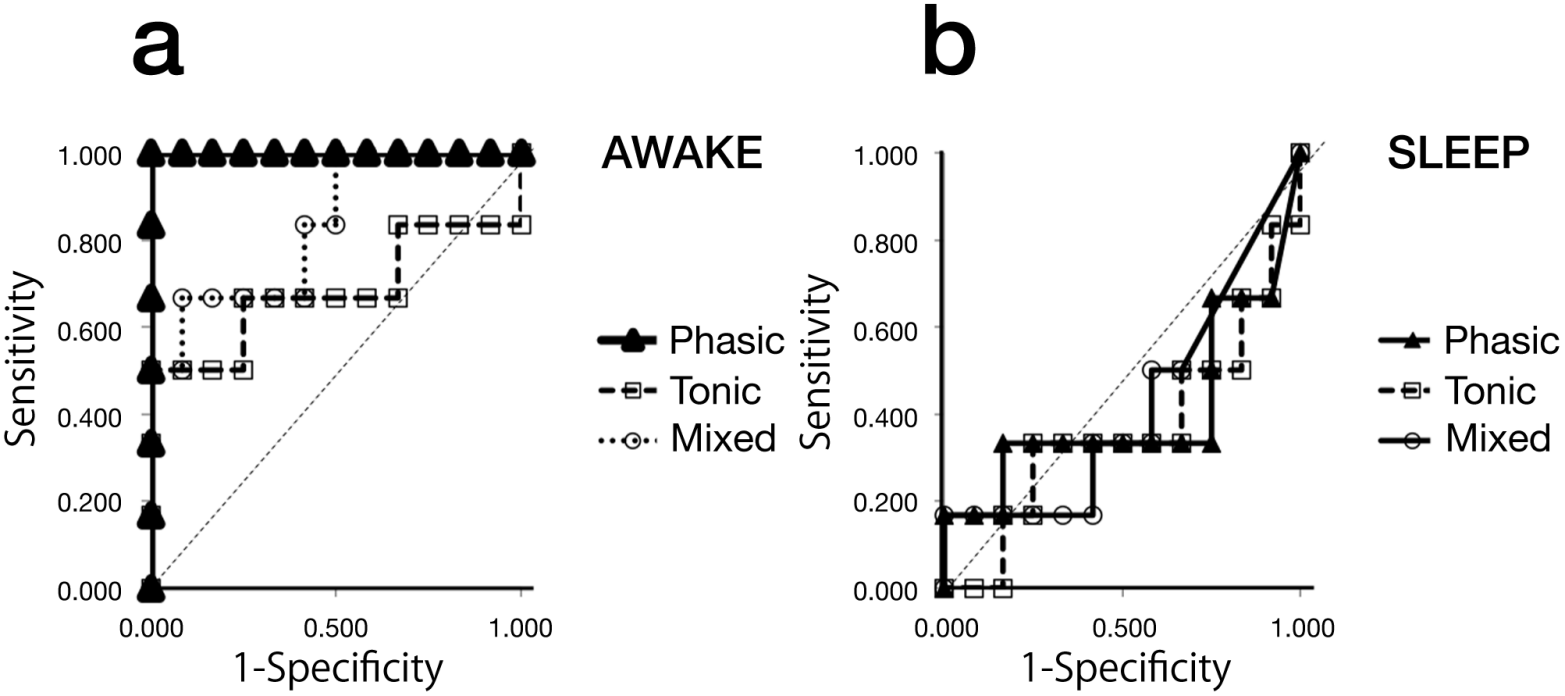
ROC curve for phasic, tonic, and mixed episodes (threshold: >20% MVC). a. ROC curve for episodes during waking hours, b. ROC curve for episodes during sleep.

Because diurnal phasic episodes seemed to be a characteristic and representative activity of the PBC group, ROC curve analysis was applied to the data to estimate a possible cut-off value for diagnosing progressive bite collapse phenomena. Figure 5 shows the ROC curve for identification of PBC group members based on the prevalence of phasic, tonic, and mixed episodes during waking hours using a 20% MVC threshold. A phasic episode incidence of 4.39/h was calculated as the most suitable cut-off value from the ROC curve. For the identification of PBC group from other two control groups using this cut-off value, sensitivity and specificity was calculated to be 1.00 and 1.00 for the samples of this study, respectively.

The results of the Student’s t-test of mean duration and mean burst strength of phasic episodes for the six subjects in each group are shown in Table 3. Regarding the properties of diurnal phasic muscle activity >20% MVC, the mean duration of the episodes were 9.0±1.2 s (range: 7.1–10.6 s) in the MC group, 12.8±5.1 s (range: 7.3–22.4 s) in the PBC group and 7.8±2.3 s (range: 4.7–10.8 s) in the YC group. The mean duration of episodes in the PBC group was significantly longer than in the MC and YC groups (*p*<0.05). The mean EMG peak activity value for 100 phasic bursts in the MC, PBC, and YC groups were 23.1±13.1% MVC, 47.0±15.6% MVC, and 26.5±13.0% MVC, respectively. The mean EMG peak activity value for the PBC group was significantly higher than for the MC and YC groups (*p*<0.05). Mean values of the inter-burst duration during phasic episodes for MC, PBC, and YC groups were 1.21±0.22 s (range: 0.89–1.55 s), 1.50±0.39 s (range: 0.97–2.10 s), and 1.18±0.16 s (range: 0.99–1.47 s), respectively. Although the mean inter-burst duration for the PBC group tended to be longer than for the other two groups, no significant difference was observed.

**Table 3:** Mean duration and mean burst strength of phasic episodes between PBC, MC and YC groups while awake and asleep.

	Mean duration (s)			Mean burst strength (%MVC)		
	PBC	MC	YC	PBC	MC	YC
AWAKE	12.8§	9.0	7.8	47.0	23.1	26.5
	(5.1)	(1.2)	(2.3)	(15.6)	(13.1)	(13.0)
SLEEP	7.3	6.7	9.9	18.7	24.1	28.0
	(5.9)	(3.8)	(4.6)	(5.9)	(3.8)	(4.6)

Mean number (S.D.).

§ *p*<0.05, vs MC and YC.

(Student’s *t*-test).

For the data during sleep, the diagnostic criteria for sleep bruxism [13] were applied to the data.

Six subjects (PBC4, PBC5, MC2, MC3, YC1, and YC2) showed more than four episodes per hour during sleep. Four of these six (PBC5, MC3, YC1, and YC2) showed more than 25 bruxism bursts per hour during sleep. Although grinding sound recording was not performed, only these four subjects could be regarded as bruxers based on EMG activity.

The normalization of the EMG activity, relative to MVC, showed that mean EMG activity during 5N bite for the MC, PBC, and YC groups was equivalent to 4.0±3.1% MVC (range: 1.5–8% MVC), 4.2±2.4% MVC (range: 1.9–8.3% MVC), and 3.1±1.2% MVC (1.3–4.6% MVC), respectively. No statistically significant differences were observed among the groups (*p* = 0.1646: f = 3.1059). Post hoc power analysis was performed to evaluate sample size. For differences in the mean incidence of phasic episodes with a threshold of 20% MVC, the subject sample size of six showed enough power (power = 0.9830) for α = 0.05, effect size = 2.41. For differences in the mean incidence of phasic episodes between the PBC and YC groups with a threshold of 20% MVC, the subject sample size of six showed enough power (power = 0.9838) for α = 0.05, effect size = 2.42.

## Discussion

Increased masticatory muscle contractions during sleep have been thought to cause occlusal overloads and thereby cause clinical complications for teeth and prostheses [2]. However, the relationship between nocturnal bruxism and dental complications remains unclear. Two major factors could explain the difficulty in revealing this relationship. One is the use of questionnaires to detect bruxism, as pointed out by the review of Manfredini et al. [4]. The actual occurrence of bruxism might not be reflected by the questionnaire data. The other possible factor is past trends in EMG recording and analysis. Because EMG studies have focused more on sleep bruxism than diurnal masticatory muscle activity, only limited knowledge has been gained about diurnal bruxism to date. In particular, no paper has reported scientific assessment of relationship between actual diurnal bruxism and periodontal disease.

In this study, we focused on the masticatory muscle activity of patients whose occlusal support is undergoing collapse. The inclusion criteria for the PBC group aimed to select patients with a progressively poor prognosis for dental treatment. As suggested by the severe resorption of the denture-supporting alveolar ridge and/or frequent loss of antagonistic teeth, the underlying forces could play a role in progression in these cases (Figure 1). None of the six subjects in the PBC group were aware of their diurnal clenching before the experiment, suggesting the difficulty in detecting diurnal bruxism using a questionnaire.

In the PBC group, only one of the six subjects was revealed to be a sleep bruxer. This unequal expression of diurnal and sleep parafunctional muscle activity could be one possible reason for the controversy about the role of sleep bruxism in the destruction of oral tissues. These findings suggest the importance of ambulatory EMG recordings to estimate the effect of masticatory muscle activity on the stomatognathic system. Although EMG studies have previously focused on nocturnal activities, EMG activity per unit of time during waking hours was shown to be significantly higher than during sleep (Figure 4). Because we spend much more time awake than asleep, the total force exerted on the stomatognathic system during waking hours has a greater impact than forces exerted during sleep.

Because all PBC group patients had complained of pain or discomfort related to denture prosthesis or residual teeth, it is reasonable to suppose that their maximal voluntary clenching would be affected by their condition. If a patient in the PBC group had difficulty clenching for the MVC task because of pain or discomfort, the standardized %MVC value could have been higher than it should have been. In the present study, all subjects achieved a standardized bite of 5N. Comparison of the %MVC value for a 5N bite among the three groups is one possible reference to check the performance of MVC. The calculated mean %MVC values for a 5N bite in the MC, PBC, and YC groups were 4.0, 4.2, and 3.1, respectively, showing no significant differences among groups. Therefore, it was inferred that the MVC procedure was performed adequately in the PBC group. However, focusing on the range of %MVC for a 5N bite, the ranges observed in the MC, PBC, and YC groups were 1.5–8% MVC, 1.9–8.3% MVC, and 1.3–4.6% MVC, respectively, suggesting that the values ranged widely. When evaluating muscle activity in patients with complicated conditions, including muscular pain and bite collapse, it is reasonable to expect difficulty in standardizing muscle activity using the maximal clench as a standard. For those cases, a low-level quantitative bite, such as a 5N bite, might be a possible reference in future studies.

Diurnal phasic episodes, unlike tonic or mixed, were observed in all PBC group subjects whose occlusal support are under collapsing progress (Figure 3). These findings lead us to surmise that diurnal phasic contraction would be highly related to the progress of bite collapse. In order to objectively utilize these findings, ROC curve analysis was applied to the data for the possible diagnose of the progressive bite collapse phenomena. As shown in the ROC curves (Figure 5), PBC group could be identified from other two groups with extremely high sensitivity and specificity, thus suggesting the future possibility to utilize the specific EMG activity for the diagnosis of the progressive bite collapse. Although the prevalence of phasic episodes was highly accurate in identifying members of the PBC group, mixed and tonic episodes might not be useful for identification [14].

The results of the present study suggest that diurnal phasic contraction of masseter muscles is a characteristic of patients with progressive bite collapse. The incidence of diurnal phasic contractions could be used for the prognostic evaluation of stomatognathic system stability.
